# Book Review

**Published:** 1935

**Authors:** 


### Reviews of Books

**Hugh Owen Thomas.** By F. Watson. Pp. xi., 94. Illustrated. London: Oxford University Press. 1934. Price 12s. 6d.—This book is mainly a sketch of the most unconventional character of the pioneer of orthopædic surgery in Great Britain. Mr. H. O. Thomas, born in 1834, was undoubtedly a searcher after Truth, and if his methods seem somewhat of the crude and rough order, it must be remembered how greatly science and knowledge in surgical matters have increased since his day. His family is thought to be descended from a Spanish boy wrecked on the coast of Anglesea, and each generation appears to have inherited a gift of "bone setting." Their fame spread, and being well-to-do yeoman farmers, they needed no remuneration when they exercised this gift. Hugh Owen Thomas was the first of the family to regularize his position by becoming a properly qualified medical man. He was followed in this course by four brothers. Dr. H. O. Thomas still gave free advice to the poor, and gave up Sundays for the treatment of patients who could not pay fees. His work and its results are to be treated in a second volume, written by an orthopædic surgeon, Mr. D. McCrae Aitken. Although never received heartily by the other surgeons in Liverpool, where he practised—though some of them consulted him for dislocations and fractures—there can be no doubt that the dock labourers and others of the poorer orders had an immense love for and trust in him. When he died in 1891 thousands of poor men and women followed him to the grave, many showing their grief by sobs and tears. He was a man of great ingenuity, devising splints and accessories as he wanted them to fit deformities and to reduce fractures. The splint devised by him for immobilization of the knee-joint was the one used during the Great War, and subsequently for fractures of the femur, and this device alone will serve to perpetuate his memory for years to come.

**The Life of Edward Jenner, M.D., F.R.S.** Second Edition. By F. D. DREWITT, M.D., F.R.C.P. Pp. xi., 151. Illustrated. London: Longmans, Green & Co. 1933. Price 6s.—This second edition of "Drewitt's Life of Jenner" reminds us that we had not the opportunity of reviewing the first edition. The life is charmingly written, and has the advantage of containing some new information from the MS. Notebook of Jenner's recently published by the Royal College of Physicians. To Gloucestershire folk and dwellers in these western shires in general the romance of Jenner's life can never grow dim. Dr. Drewitt brings him clearly before us—the nature-lover, the artist, the family doctor and the keen scientist all combined within the compass of a single human mind and body. Bristolians will note with surprise one omission. Mention is made of various prisoners of war released by Napoleon at Jenner's request: the name of Francis Gold, who kept an anatomy school in College Green, Bristol, is not included, even though Gold published the details of his release in the preface to his translation of Ramond's *Travels in the Pyrenees.*

**Biological Politics.** By F. W. INMAN, M.B. Pp. xi., 258. Bristol: John Wright & Sons Ltd. 1935. Price 7s. 6d.—A reading of this little book gives one an impression such as one experiences when taking a country walk with a well-read, thoughtful, chatty elderly gentleman, who discourses as he walks, turning from subject to subject as fresh objects of interest come into sight. The general theme is the parallels that may be drawn from biology, especially human biology, and the modern political situation. He has views with some of which we concur, but not all. The doctor who likes a small volume of popular interest to wile away a few leisure hours may well obtain and read Dr. Inman's contribution.

**A Synopsis of Surgical Anatomy.** Second Edition. By A. L. McGREGOR, M.Ch. Pp. xvii., 644. Illustrated. Bristol: John Wright & Sons Ltd. 1934. Price 17s. 6d.—That this book fills a distinct need seems to be indicated by the fact that a second edition has been called for only two years after the first. A book of 644 pages, it contains an enormous amount of information, much more anatomical than surgical, and so rather strong meat for a students' text-book. Applied anatomy is not a popular subject with the student at a time when his energies are devoted more particularly to clinical subjects, and it is almost too much to expect that he will make a close study of so monumental a work as this. A smaller book is, quite naturally, more to his taste, and a few illustrations here and there of the way the anatomical facts presented are applied makes such a book more acceptable. Having said that, it must be said also that the facts are here presented in an eminently readable and assimilable form. In fact, it is possible to pick up the book, open it at random, and find at once something interesting, and presented in a particularly attractive way. Even the student will find, if he does not feel disposed to read it systematically from cover to cover, that nevertheless it is far from profitless to dip into at intervals, even if only for the elucidation of some anatomical problem which troubles him at the moment. The illustrations, simple but adequate, add to the value of the book, and the index is complete and accurate.

**What of the Child?** By A. KEFALAS, M.B. Pp. xvi., 187. London: William Heinemann, Ltd. 1934. Price 5s.—Dr. Kefalas has written a sensible and helpful book on the development and guidance of children, both physical and mental. The author's views on punishment, religious instruction and sex information are very well balanced. His advice on the choice of schools is delightful. At the outset he predicates two kinds to be specially avoided, "the Scholarship-Miracle Producer" and the "Forcing Ground for useful Subordinates." His own religious convictions are not unduly prominent, though we get an inkling from one of the closing sentences of the book, "the God of the Cross and the Gothic Arch is the God of the Microscope and the Workshop too." A wholly commendable book.

**A Practical Manual of Diseases of the Chest.** By M. DAVIDSON, M.D. Pp. xii., 528. Illustrated. London: Oxford University Press. 1935. Price 42s.—As the title suggests, the aim of the book is essentially practical. Academic discussion is subordinated to clinical considerations and treatment. The opening section, which deals with anatomical and physiological details, is beautifully illustrated, and radiology of the normal chest receives adequate attention. The book is excellently arranged, and every section contains an account of the recent advances in each particular subject. Where all is so good it is difficult to pick out individual items, but the sections on bronchiectasis, asthma, diseases of the pleura, and industrial diseases certainly merit special attention. The author devotes much space to pulmonary tuberculosis; this portion of the book is perhaps outstanding, and the reader feels that the views expressed are the product of ripe clinical experience. The section on intrathoracic new growths is excellent, and again shows the author's particular interest and personal experience. Throughout the book is lucid and easy to read. The illustrations, more particularly the radiograms, are of a very high order. There is hardly any problem in the range of diseases of the lungs and pleura which is not dealt with in a way calculated to help both consultant and general practitioner. The author is to be congratulated on producing the most useful book on diseases of the chest that has appeared for many years.

**Baths and Medicinal Waters of Britain and Europe.** By M. G. FOSTER, O.B.E., M.D. Pp. viii., 225. Illustrated. Bristol: John Wright & Sons Ltd. 1933. Price 12s. 6d.—Shortly before his lamented death Dr. Michael Foster had completed a survey of the baths and spas of Great Britain for the information of general practitioners. Since Sir Herman Weber's work on climatotherapy and balneotherapy appeared no comparable book has been published in England. Dr. Foster's experience was wide, and he wrote with ease and brevity that is most enviable. This volume gives in a small compass all the essential facts about the British waters and the claims made for their healing virtues. It will be wise to check these claims by occasional reference to the experimental data contained in Von Noorden's *Metabolism and Practical Medicine*, where the proofs of the accuracy of these claims are critically examined. One regrets that not even in the preface nor the introductory "historical" chapter is mention made of the masterly survey contained in Von Noorden's inexpugnable work.

**X-Ray Interpretation.** By H. C. H. BULL, M.A., M.B. Pp. xxxiv., 382. Illustrated. London: Oxford University Press. 1935. Price 21s.—The author indicates that the object of this book is to relate the X-ray appearances of various pathological conditions in all parts of the body with the actual lesions of gross pathology. He has described in twenty chapters, covering the various sections of the body in turn, the normal and the more common pathological conditions which are capable of demonstration by X-rays, and has wisely illustrated the text by 280 excellent line drawings, which serve their purpose much better than reproductions of actual skiagrams ; in fact, the illustrations constitute the best part of the book, and the type and general arrangements are also good. Its matter is fairly up to date, and on the whole sound, though there are some important omissions, and the chapter on the radiology of the chest is poor compared with those on the intestinal and genito-urinary tracts. Terminology employed is on the whole accurate, but one gets rather tired of the misuse of the word "fundus" in relation to the stomach. There is a useful short chapter on dental radiology and a good index. Apart from the illustrations there is nothing original in the book, and the author would probably have attained his object if he had included illustrations of actual pathological conditions side by side with their X-ray appearances. Such a book is yet to be written, and is much needed. Meanwhile, the present volume will be found useful to students and others as a handbook of reference.

**Principles in the Treatment of Inflammation.** By T. E. Hammond. Pp. xii., 209. London: H. K. Lewis & Co. Ltd. 1934. Price 10s. 6d.—The thesis propounded by the author is that there may be distinguished five inflammatory states : acute sthenic, acute asthenic, chronic sthenic, chronic asthenic and septicæmic. The reaction varies with the type of bacterial infection, and generally gram positive organisms cause sthenic and gram negative asthenic inflammation. If one agrees with these pronunciamentos one will perhaps like the book.

**Injuries and their Treatment.** By W. E. Tucker, B.Ch. Pp. xi., 173. Illustrated. London: H. K. Lewis & Co. Ltd. 1935. Price 9s.—This book is of special interest to those engaged in the physio-therapeutic departments and to practitioners who treat cases of injuries. The author points out that injuries should be energetically treated, and not left to nature to recover ; he shows that by the various forms of treatment at our disposal we may reduce the time of disablement by many weeks. The treatment of the commoner injuries is outlined, and the indications for the various forms of physio-therapy are plainly stated. Special mention is made of the value of manipulative treatment, the indications and principles of which are explained, the text being aided by clear drawings and excellent photographs.

**A Short Practice of Surgery.** Second Edition. By H. BAILEY, F.R.C.S., and R. J. McN. LOVE, M.S., F.R.C.S. Pp. viii., 987. Illustrated. London : H. K. Lewis and Co. Ltd. 1935. Price 30s.—This is an excellent guide to the student of surgery. It is both lucid and interestingly written, and the authors have compressed all that is important and up to date in a remarkably small space. There is scarcely an irrelevant word in the book. A distinctive feature and extremely helpful is the frequent classification of causes, pathological sequelæ and treatment of different diseases. The profuse illustrations are also a meritorious part of the work ; perhaps a few of them might be a little less sketchy, for the general standard of the book is so excellent that the extra trouble and cost entailed would be fully justified. For value and accuracy the book is unsurpassed.

**The Treatment of Common Female Ailments.** Third Edition. By F. J. MCCANN, M.D., F.R.C.S. Pp. viii., 379. London : Edward Arnold and Co. 1934. Price 12s. 6d.—The subject-matter has been brought up to date in the third edition of this text - book ; it has been written by a gynæcologist of wide experience, and is largely a record of his own observation and practice. Primarily intended for the general practitioner, this book can also be read with profit by the student of medicine. In order to reduce the incidence of chronic ill-health amongst parous women, particular stress is laid on the importance of preventive measures, and on the need for adequate surgical treatment where such injuries occur. This book can be recommended as a useful addition to the medical practitioner's library.

**Anæsthesia in Labour.** By KATHARINE G. LLOYD-WILLIAMS, M.D. Pp. 96. Illustrated. London : Edward Arnold and Co. 1934. Price 5s.—Miss Lloyd-Williams is to be congratulated on her summary of modern methods in anæsthesia in midwifery. Apparently a method yet remains to be found which is completely reliable, and the general practitioner attending a case in a private house with one nurse and many anxious relatives will probably still remain faithful to chloroform, ether, hyoscine and morphia.

